# Screening and early therapeutic intervention of bonding disorders at first six months of life: An alternative to prevent disorganised attachment and severe mental disorder

**DOI:** 10.1192/j.eurpsy.2023.286

**Published:** 2023-07-19

**Authors:** G. Hernández-Santillán, M. Alcamí-Pertejo, B. Palacios-Hernández, G. Lahera-Forteza, A. Fernández-Sánchez, M. F. Bravo-Ortiz

**Affiliations:** 1 Medicine and Surgery Doctorate Programm, Universidad Autónoma de Madrid; 2 Children & Adolescent Psychiatry, Hospital Universitario Príncipe de Asturias; 3 Children & Adolescent Psychiatry, Hospital Universitario La Paz, Madrid, Spain; 4 Mental Health Laboratory, Universidad Autónoma del Estado de Morelos, Morelos, Mexico; 5 Universidad de Alcalá; 6 Psychiatry, Hospital Universitario Príncipe de Asturias; 7 Hospital Universitario La Paz; 8 Universidad Autónoma de Madrid; 9 Psiquiatría, Hospital Universitario La Paz, Madrid, Spain

## Abstract

**Introduction:**

Disorganized attachment has been described as an important risk factor for developing serious mental disorders in childhood, adolescence and adulthood, such as borderline personality disorder, psychoses, afective disorders, and a higher suicide risk, for instance. Bonding disorders (BD) in parents are related to insecure and disorganized attachment in children. BD can be early diagnosed at 4 to 6 weeks after birth.

**Objectives:**

Determine if there is a significant difference between the results of the prevalence of affective disorders, disorganized attachment, and suicidal risk five years after the birth of the offspring of parents with and without attachment disorders detected in the first year postpartum during the covid-19 pandemic.

**Methods:**

Describe a pilot project of an analytical prospective study following a cohort of parents from the cohort SAMPECO/PEMHSCO (Perinatal Mental Health in Spain during the Covid-19 pandemic). The cohort is planned to be divided into two groups: with bonding disorders an without bonding disorders, which was established using the Postpartum Bonding Questionnaire (Brockington, 2006). Follow the offspring of both groups for 5 years and compare the results of disorganized attachment, affective disorders and suicide risk.

**Results:**

The cohort SAMPECO/PEMHSCO was recruited between March 2021 and June 2022. There was measured postpartum depression in mothers and fathers using the EPDS and bonding disorders in parents using the PBQ validated to the Spaniard population. More than 1500 families were involved at the beginning and around 450 families finished the follow-up six months after birth. Around 500 families were lost because of non-right contact information.

**Conclusions:**

The covid-19 pandemic has seriously affected the mental health of the general population. Consequently, there is a higher demand for mental health assistance by public and private sanity sectors. Currently, the youth population is suffering very much from the consequences of isolation and other social factors, and many families who had babies in this period haven’t had enough support to breed and look after both their babies and themselves. Some papers suggest that the prevalence of perinatal mental disorders in parents has increased since the covid-19 pandemic because of several factors. Paradoxically, despite the high preventive potential of early intervention in the perinatal period, there are not yet exist well-equipped perinatal mental health units to solve this problem. It is urgent to boost the development of Perinatal Mental Health Services to prevent a major worsening of the situation and to prevent the increasing rate of severe mental disorders in children, adolescents and adults.

**Disclosure of Interest:**

None Declared

